# Bi-Atom Electrocatalyst for Electrochemical Nitrogen Reduction Reactions

**DOI:** 10.1007/s40820-021-00638-y

**Published:** 2021-04-07

**Authors:** Wenchao Zhang, Bin-Wei Zhang

**Affiliations:** 1 Institute of Environmental Engineering, School of Metallurgy and Environment, Central South University, Changsha, 410083 Hunan People’s Republic of China; 2 Chinese National Engineering Research Center for Control and Treatment of Heavy Metal Pollution, Changsha, 410083 Hunan People’s Republic of China; 3 Institute for Superconducting and Electronic Materials (ISEM), School of Mechanical, Materials, Mechatronics and Biomedical Engineering, Faculty of Engineering and Information Sciences, University of Wollongong, Wollongong, NSW 2500 Australia

**Keywords:** Electrochemical nitrogen reduction reaction, Bi-atom catalysts, Excellent activity, High selectivity

## Abstract

The electrochemical nitrogen reduction reaction (NRR) to directly produce NH_3_ from N_2_ and H_2_O under ambient conditions has attracted significant attention due to its ecofriendliness. Nevertheless, the electrochemical NRR presents several practical challenges, including sluggish reaction and low selectivity. Here, bi-atom catalysts have been proposed to achieve excellent activity and high selectivity toward the electrochemical NRR by Ma and his co-workers. It could accelerate the kinetics of N_2_-to-NH_3_ electrochemical conversion and possess better electrochemical NRR selectivity. This work sheds light on the introduction of bi-atom catalysts to enhance the performance of the electrochemical NRR.

## Highlights

- A new heteronuclear bi-atom electrocatalyst has been proposed by Ma and his co-workers.
- The FeV@C_2_N bi-atom electrocatalyst achieved excellent electrochemical NRR performance.
- The FeV@C_2_N bi-atom electrocatalyst could effectively suppress the side and competing HER reaction, and thus possess better electrochemical NRR selectivity.

## Graphical abstract

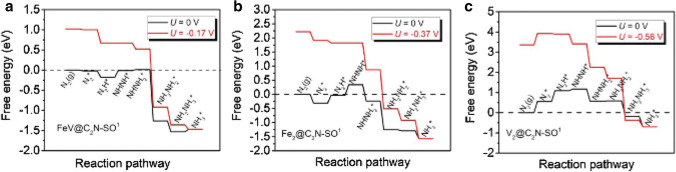

The electrochemical nitrogen reduction reaction (NRR) to directly produce NH_3_ from N_2_ and H_2_O under ambient conditions has attracted significant attention due to its ecofriendliness compared with the traditional Haber–Bosch process [1, 2]. Nevertheless, the electrochemical NRR presents several practical challenges, including sluggish reaction and low selectivity [3, 4]. The slow kinetics is caused by the extremely strong N≡N triple bond (941 kJ mol^−1^) and the great energy gap between highest occupied molecular orbital (HOMO) and the lowest unoccupied molecular orbital (LUMO) of the N_2_ molecule [5]. The hydrogen evolution reaction (HER) is the main side reaction responsible for the low selectivity, which shares a very close potential window with the NRR in both alkaline and acidic electrolytes [6, 7]. Fortunately, the electrochemical NRR depends heavily on its electrocatalysts [8–10]. Therefore, advanced rational design of the electrochemical NRR electrocatalysts to achieve outstanding performance and high selectivity is urgently required [11–13]. Various NRR electrocatalysts, including metal-free catalysts, single-atom catalysts, metal nanomaterials, nitrides/oxides/sulfides/carbides, etc*.*, have been reported with the aim of high NH_3_ yield since 2016 [14]. Nevertheless, a promising candidate, a heteronuclear bi-atom electrocatalyst, has been little studied for the electrochemical NRR.

Recently, Ma and co-workers [15] designed a new heteronuclear bi-atom electrocatalyst, Fe, V co-doped C_2_N (FeV@C_2_N), to accelerate the kinetics of the NRR and suppress the hydrogen evolution reaction (HER), which occurs as a side reaction. This FeV@C_2_N electrocatalyst achieved excellent electrochemical NRR performance. The nitrogenated holey structures in C_2_N could anchor these Fe and V atoms; additionally, the unoccupied/occupied *d* orbitals of Fe and V atoms may accept/donate electrons from/to N_2_ (Fig. 1a). Therefore, Fe and V atoms could be stable on the C_2_N matrix and serve as active sites to electrocatalytically transform N_2_ into NH_3_. The FeV@C_2_N could weaken the N≡N triple bond and increase the Bader charge difference of two chemisorbed N atoms, as shown in Fig. 1b−d. More importantly, the FeV@C_2_N possesses the greatest ability to activate N_2_ compared to Fe_2_@C_2_N and V_2_@C_2_N.

**Fig. 1:**
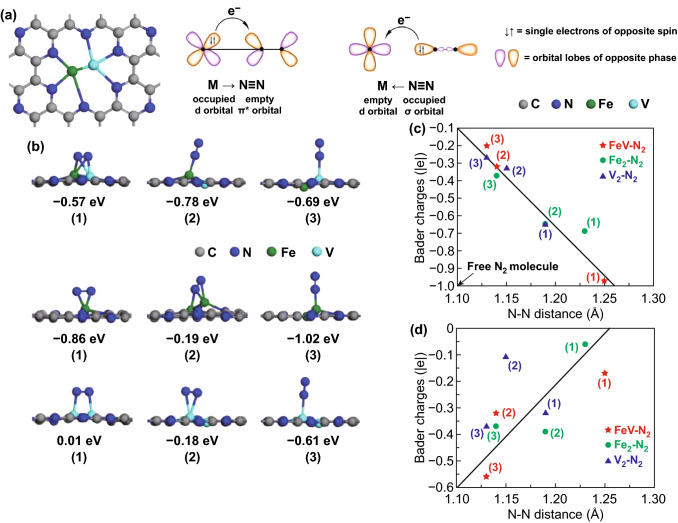
**a** Optimized structures of FeV anchored on C_2_N substrate and simplified schematic diagram of the bonding between the transition metal and N_2_. **b** Optimized structures and corresponding adsorption energies of N_2_ adsorption on FeV@C_2_N, Fe_2_@C_2_N, and V_2_@C_2_N. **c** Relationship between Bader charges of adsorbed N_2_ and N–N bond lengths. **d** Relationship between Bader charge difference of two adsorbed N atoms and N–N bond lengths [15]. Copyright 2020 Elsevier

Furthermore, Ma and co-workers [15] proposed the mechanism of N_2_ reduction and free energy diagrams on side-on configurations of FeV@C_2_N, Fe_2_@C_2_N, and V_2_@C_2_N, and they believe that FeV@C_2_N is the most promising electrocatalyst for the NRR compared with the other two. There are only two reaction steps from N_2_H^*^ to NHNH^*^ and NHNH^*^ to NHNH_2_^*^, which are endothermic for FeV@C_2_N; thus, these two steps are the potential-determining step (PDS) with free energy of 0.17 eV, as shown in Fig. 2a. The Fe_2_@C_2_N shares the same PDS but with a higher free energy of 0.37 eV (Fig. 2b). In V_2_@C_2_N, the PDS is the formation of N_2_^*^, with the greatest free energy of 0.56 eV (Fig. 2c). The HER, as a competing and side reaction, was also investigated for these three samples. The calculated results indicated that FeV@C_2_N and Fe_2_@C_2_N have better NRR selectivity. V_2_@C_2_N, because of the competing HER, is not a good candidate for the electrochemical NRR.

**Fig. 2:**
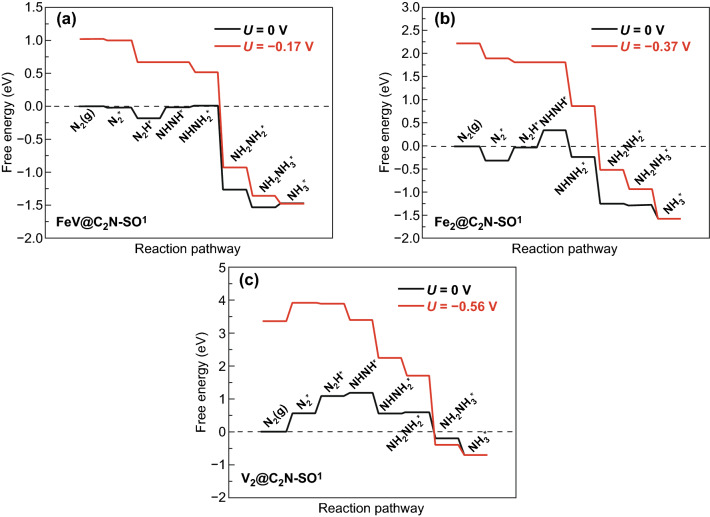
**a**–**c** Free energy diagrams of the three electrocatalysts [15]. Copyright 2020 Elsevier

In sum, Ma and co-workers proposed FeV@C_2_N as an outstanding heteronuclear bi-atom electrocatalyst for the electrochemical NRR, with high activity and better selectivity. It could enhance the kinetics of N_2_-to-NH_3_ electrochemical conversion with a low potential PDS of -0.17 V. Moreover, this FeV@C_2_N electrocatalyst could effectivity suppress the side and competing HER reaction, and thus possess better electrochemical NRR selectivity. This work sheds light on the introduction of heteronuclear bi-atom electrocatalysts to enhance the performance of the electrochemical NRR and opens a new way to understand the electrochemical NRR mechanism.

In the future, two possible prospects could be effective approaches to optimize the electrocatalysts with the aim of improved NRR activity and selectivity, and reveal the mechanisms of the electrochemical NRR as well. Firstly, theoretical calculations could be employed to predict potential NRR electrocatalysts and provide various types of optimization guidance to the experiments. For example, high-throughput computing can identify the poisoning and decomposition of electrocatalysts under electrochemical conditions, including the pH and the electrolyte effect, which can provide a deeper insight into the mechanism under real operation conditions. On the other hand, advanced characterization, including *in-situ* and *operando* atomic-resolution transmission electron microscopy and X-ray absorption spectroscopy, can be developed to identify the real active sites and composite evolution of the electrical double layer. With the significant efforts that have been made in the past few years, the electrochemical NRR appears promising to replace the traditional Haber–Bosch process to produce NH_3_. Nevertheless, a reproducible and excellent electrochemical NRR catalyst is still expected to be proposed as a standard electrocatalyst, due to the doubt that has arisen on the actual NRR performance. A benchmarking protocol to accurately quantify the electrochemical NRR activity and selectivity should be established. We believe that, with much effort, the fundamental issues and technological drawbacks will be addressed in the not-too-distant future, and the electrochemical NRR can play an important role in NH_3_ yield.
